# Variation in Hygienic Behavior and Its Associations with Colony-Performance and Wing Morphometric Traits Among Honey Bee Colonies from Five Apiaries in Kazakhstan

**DOI:** 10.3390/insects17100978

**Published:** 2026-09-22

**Authors:** Ulzhan Nuraliyeva, Gaukhar Moldakhmetova, Gulim Khalykova, Merey Torekhanov, Aksholpan Shokan, Akbota Taufikh, Maxat Toishimanov

**Affiliations:** 1 Kazakh Research Institute of Livestock and Fodder Production, Almaty 050035, Kazakhstan; 2 Zooengineering and Biotechnology Department, Kazakh National Agrarian Research University, Almaty 050010, Kazakhstan; 3 Institute of Genetics and Physiology, 93 Al-Farabi Avenue, Almaty 050060, Kazakhstan; 4 Food and Environmental Safety Laboratory, Kazakh National Agrarian Research University, Almaty 050010, Kazakhstan; 5 Department of Food Safety and Quality, Faculty of Food Technology, Almaty Technological University, Almaty 050012, Kazakhstan

**Keywords:** *Apis mellifera*, hygienic behavior, pin-killed brood assay, morphometry, cubital index, colony performance, honey bee breeding

## Abstract

Honey bee hygienic behavior is an important colony-level defense trait with potential relevance to selective breeding. The aim of this study was to evaluate variation in hygienic behavior among honey bee colonies from five apiaries in Kazakhstan and to examine its associations with colony-performance traits and wing morphometric characteristics. Hygienic behavior was assessed in 50 colonies using the pin-killed brood assay, with brood removal recorded 24 h after treatment. Significant differences in hygienic behavior were observed among the five studied apiaries (*p* < 0.001), with the highest mean brood-removal efficiencies recorded in the Nurik and KazNARU apiaries and the lowest in the Baizhigitov apiary. Principal component and hierarchical clustering analyses revealed multivariate differentiation among colonies based on behavioral, performance, and morphometric characteristics. Although the cubital index was strongly correlated with hygienic behavior in the pooled analysis, the within-apiary association was not statistically significant after accounting for apiary identity (β = 9.42, 95% CI: −2.82 to 21.65, *p* = 0.128), indicating that the overall relationship was primarily attributable to differences among apiaries. However, because apiary identity is associated with ecological conditions as well as potentially differing management practices and honey bee population backgrounds, these patterns should not be interpreted as independent causal effects of ecological conditions. The findings provide baseline information on variation in hygienic behavior and associated phenotypic traits in honey bee colonies from Kazakhstan and support further studies incorporating broader environmental, management, and genetic characterization before their potential application in selective breeding programs.

## Simple Summary

Honey bee colonies are threatened by numerous diseases and parasites, making the identification of naturally resilient colonies important for sustainable beekeeping. Hygienic behavior is a colony-level defense mechanism in which worker bees detect and remove unhealthy or dead brood. In this study, we evaluated hygienic behavior in 50 honey bee colonies from five apiaries in Kazakhstan and examined its associations with colony-performance traits and wing morphometric characteristics. Substantial differences in brood-removal efficiency were observed among the studied apiaries, with the highest mean values recorded in the Nurik, KazNARU, and Zhusip apiaries and the lowest in the Baizhigitov apiary. Multivariate analyses revealed associations among hygienic behavior, colony-performance traits, and morphometric characteristics. Although the cubital index showed a strong overall association with hygienic behavior, this relationship was not significant after accounting for within-apiary variation, indicating that the overall association was largely attributable to differences among apiaries. Because ecological conditions were partly confounded with apiary identity, management practices, and honey bee population background, these findings should be interpreted as associations rather than independent ecological effects. The study provides baseline information for future evaluation of hygienic behavior and related phenotypic traits in honey bee populations of Kazakhstan and may support further breeding studies incorporating broader environmental, management, and genetic characterization.

## 1. Introduction

The western honey bee (*Apis mellifera* L.) is the most economically important managed pollinator worldwide, underpinning the productivity of a large share of insect-pollinated crops and contributing substantially to global food security and ecosystem stability [1]. Over the past two decades, however, managed colonies have experienced elevated and geographically widespread losses driven by the combined action of pathogens, parasites, pesticide exposure, and environmental stressors [2]. Foremost among these threats is the ectoparasitic mite *Varroa destructor* and the viral complex it transmits, which together constitute the single greatest cause of colony mortality in most beekeeping regions [3]. Because chemical acaricides and antibiotics provide only temporary control and increasingly select for resistance, sustainable apiculture depends on identifying and propagating honey bee stocks expressing heritable resistance traits, including hygienic behavior and Varroa-sensitive hygiene.

Among these mechanisms, hygienic behavior is one of the most extensively documented and agriculturally valuable forms of social immunity in honey bees [4]. It describes the ability of worker bees to detect, uncap, and remove diseased, dead, or parasitized brood from sealed cells before a pathogen or parasite can complete its developmental cycle and spread through the colony [5]. First characterized genetically by Rothenbuhler in his classic studies of nest-cleaning responses toward disease-killed brood [6], the trait is now recognized as a colony-level defense that complements the individual and physiological immune responses of the superorganism [7]. Hygienic behavior is a heritable, polygenic colony-level trait that can be evaluated and incorporated into breeding programs [8]. The foundational syntheses of Spivak and Gilliam established its central role in resistance to brood diseases and mites and consolidated the empirical basis for its application in colony health management [9,10].

Hygienic behavior contributes to colony defense against several brood pathogens and parasites, including *Ascosphaera apis*, *Paenibacillus larvae*, and *Varroa destructor* [11,12,13,14]. A specialized form, Varroa-sensitive hygiene (VSH), involves the detection and removal of brood infested by reproductive mites [15,16]. However, general hygienic behavior measured using killed-brood assays and VSH represent related but distinct phenotypes and should not be considered interchangeable. Field studies confirm that colonies selected for hygienic behavior sustain lower mite loads and reduced disease pressure under commercial conditions [17], establishing the trait as a cornerstone of integrated, low-input colony health management. However, general hygienic behavior measured by removal of experimentally killed brood and Varroa-sensitive hygiene are related but distinct phenotypes, and performance in a pin-killed brood assay should not be interpreted as a direct measurement of VSH or Varroa resistance.

Quantifying hygienic behavior in a standardized and repeatable manner is essential for both research and selective breeding. The most widely adopted approaches are the pin-killed and freeze-killed brood assays, in which a defined area of sealed brood is experimentally killed and the proportion of cells cleaned within a fixed interval is recorded [18,19]. Colonies removing a high proportion of experimentally killed brood within 24 h are generally considered to express strong hygienic behavior and may represent candidates for further evaluation in breeding programs [20]. These assays have been incorporated into standardized protocols for evaluating hygienic behavior in honey bee colonies [21,22], while refinements of the recording methodology have enabled estimation of the underlying genetic parameters [23]. Reported heritability estimates for hygienic behavior vary among populations and methodologies, ranging from approximately 0.18–0.36 in some studies to substantially higher estimates in selected populations [24,25,26] and have supported the establishment of dedicated breeding lines and coordinated pan-European and North American programs selecting for hygienic behavior and *Varroa* resistance [27,28,29,30].

Wing morphometry provides a practical means of assessing phenotypic population structure because forewing venation traits differ among honey bee evolutionary lineages, subspecies, and admixed populations [31]. Classical diagnostic indices derived from forewing venation—most notably the cubital index, together with the discoidal shift and the Hantel index—have long been used to differentiate *A. mellifera* lineages [31]. The advent of computer-assisted and geometric morphometric methods, including landmark-based analysis of wing venation and dedicated software, has increased the resolution and objectivity of honey bee population characterization [32,33]. Geometric morphometric approaches can discriminate among subspecies and identify introgressed or hybrid populations [34,35,36], making wing morphometry a useful complement to behavioral and genetic characterization. In the context of hygienic behavior, morphometric traits are relevant primarily as indicators of population and subspecies structure rather than as traits expected to directly determine brood-removal behavior. Because hygienic performance may also vary among honey bee genetic backgrounds, joint analysis of morphometric and behavioral traits can help determine whether variation in hygienic behavior covaries with phenotypic population structure. Such associations, however, should not be interpreted as evidence of a direct functional effect of wing morphology on hygienic behavior.

Both the behavioral and the morphometric traits of honey bees are shaped by the environments in which populations evolve and are maintained. Honey bee subspecies are distributed along well-defined ecological and geographical gradients, and conserving this locally adapted diversity is increasingly recognized as a priority for resilient apiculture [37,38]. Spatial analyses have repeatedly shown that morphometric characters vary clinally with climate, altitude, and latitude [39,40], reflecting adaptation to regional conditions. Genotype-by-environment studies further demonstrate that colony survival, behavior, and performance may depend jointly on genetic origin and local environmental conditions [19,41], emphasizing the importance of considering both population background and environmental context when evaluating colony phenotypes. These observations suggest that hygienic behavior, colony-performance traits, and morphometric characteristics may covary among honey bee populations maintained under different ecological conditions. However, such covariance may reflect interacting genetic, environmental, and management factors rather than an independent ecological effect.

Kazakhstan, whose territory spans foothill, mountain, and steppe landscapes across Central Asia, harbors honey bee populations of the introduced *A. m. carnica* coexists with local and admixed honey bee stocks [42,43]. Recent morphometric and molecular studies have begun to characterize the diversity and population structure of honey bees in Kazakhstan [44], but information integrating hygienic behavior, colony-performance traits, and wing morphometry remains limited. Therefore, the present study aimed to (i) evaluate variation in hygienic behavior, measured as the removal of pin-killed brood within 24 h, among colonies from five apiaries in Kazakhstan; (ii) characterize the associated colony-performance and wing-morphometric traits; and (iii) examine multivariate and colony-level associations between hygienic behavior and these phenotypic characteristics while accounting for the clustering of colonies within apiaries. The study was designed to characterize general hygienic brood-removal behavior and did not directly assess VSH, *Varroa destructor* infestation, pathogen load, or colony survival.

## 2. Materials and Methods

### 2.1. Study Area and Experimental Apiaries

The study was conducted from May to July 2026, during the active beekeeping season in five commercial and educational apiaries located in different natural and climatic zones of Kazakhstan, including foothill, mountain, and steppe regions. All apiaries included in the study were stationary. The study included colonies maintained by local beekeepers and representing *A. m. carnica* and locally maintained honey bee populations. However, subspecies identity was not genetically verified for individual colonies; therefore, the population background should be regarded as beekeeper-reported or locally recognized rather than genetically confirmed.

The geographical location, ecological category, total number of colonies, number of sampled colonies, and production direction of the five studied apiaries are summarized in Table 1.

### 2.2. Experimental Colonies

A total of 50 *Apis mellifera* colonies were included in the study, with 10 colonies selected from each of the five stationary apiaries. Colonies were required to be queen-right, with a naturally mated and actively laying queen. The exact age of the queens was not systematically recorded and therefore could not be included as a colony-selection criterion. Queen age, pedigree, and genetic origin were not standardized among apiaries; therefore, potential queen-related variation could not be separated from other apiary-level effects.

Prior to inclusion, colony strength was assessed by frame-by-frame visual inspection based on the adult bee population and the amount and distribution of worker brood. Colonies were considered broadly comparable when they had a well-established adult worker population, sufficient open and sealed worker brood, adequate food reserves, and no obvious signs of marked weakness or brood disease. Colonies were not experimentally equalized by transferring brood, adult bees, or food reserves among colonies; rather, colonies of broadly comparable strength were selected under their existing field conditions.

For inclusion in the hygienic-behavior assay, each colony was required to contain at least one brood comb with a continuous area of healthy sealed worker brood sufficient to select approximately 100 capped worker-brood cells for the standardized PKB assay. Colonies that were queenless, markedly weak, or showed visible clinical signs of brood disease were excluded. From the colonies meeting these criteria, 10 colonies per apiary were selected for evaluation. The use of an equal number of colonies from each apiary provided a balanced sampling design and equal representation of the five study apiaries.

### 2.3. Assessment of Hygienic Behavior

Hygienic behavior was assessed at the colony level using the pin-killed brood (PKB) assay, following the general approach described by Newton and Ostasiewski [18]. The same standardized procedure was applied to all 50 experimental colonies.

The assay was conducted from May to July 2026, corresponding to the active beekeeping season. For each colony, a brood comb containing a continuous area of healthy sealed worker brood was selected. An area containing approximately 100 sealed worker-brood cells was marked, and each selected cell was pierced through the center of the wax capping with a sterile entomological pin to kill the underlying brood. The treated comb was subsequently returned to its original position in the brood nest.

After 24 h, the marked brood area was re-examined and the number of cells from which the killed brood had been completely removed was recorded. A cell was considered completely cleaned only when the dead brood had been entirely removed; cells that were only uncapped or still contained visible brood remains were not counted as completely cleaned.

The PKB assay was performed once per experimental colony. All colonies were assessed using the same procedure and the same 24 h observation interval. All assessments were performed and scored by the same trained observer to minimize observer-related variation. An untreated control brood area was not evaluated in parallel with the pin-killed area.

Hygienic behavior (HB) was calculated for each colony as the percentage of pin-killed brood completely removed within 24 h:

Hygienic behavior (%) = (Number of completely cleaned cells/Total number of pin-killed cells) × 100

The resulting percentage was used as the colony-level measure of hygienic behavior in all subsequent statistical analyses. The PKB assay in the present study was used specifically as a measure of general hygienic brood-removal behavior and was not considered a direct measurement of VSH, *Varroa destructor* infestation, pathogen resistance, or colony survival. For descriptive interpretation, colonies were classified according to the percentage of pin-killed brood removed within 24 h as highly hygienic (≥95%), moderately hygienic (85–<95%), or low hygienic (<85%). Classification was applied at the individual-colony level rather than to apiary means (Figure 1).

### 2.4. Colony Performance Evaluation

Colony-performance traits were evaluated under routine field conditions. Calmness, gentleness, and swarming tendency were assessed using a four-point scoring system (1–4) based on standardized honey bee colony performance-testing criteria [22], in which higher scores represent more favorable expression of the respective trait. Colony productivity and overwintering performance were also recorded as comparative four-point scores as described below, whereas winter feed consumption was recorded quantitatively and expressed in kilograms per colony.

Calmness was evaluated during routine colony inspection according to the movement and response of worker bees on the comb. A score of 1 indicated pronounced running or dispersal of bees from the comb, 2 indicated partial movement away from the comb surface and clustering toward the edges, 3 indicated movement of bees while remaining on the comb, and 4 indicated calm behavior, with bees remaining stable on the comb during handling.

Gentleness was evaluated according to the defensive response of worker bees during routine colony inspection. Scores ranged from 1 (strong defensive response during handling) to 4 (very gentle behavior with minimal defensive response), with scores of 2 and 3 representing intermediate levels of defensiveness.

Swarming tendency was evaluated from signs of preparation for swarming observed during colony inspection. A score of 1 indicated active or very strong swarming tendency, 2 strong swarming tendency with advanced signs of preparation for swarming, 3 low swarming tendency, and 4 no evident swarming tendency.

Overwintering performance was evaluated after the overwintering period using a comparative four-point scale based on the general post-winter condition of the colony and preservation of colony strength. Scores ranged from 1 (poor overwintering performance) to 4 (very good overwintering performance).

Colony productivity was evaluated comparatively on a four-point scale based on honey production relative to other colonies maintained under the same apiary conditions. A score of 1 represented comparatively low productivity, 2 below-average productivity, 3 above-average productivity, and 4 comparatively high productivity. Thus, this variable represents a comparative productivity score rather than absolute honey yield expressed in kilograms.

Winter feed consumption was recorded quantitatively and expressed in kilograms per colony. Colonies were overwintered primarily on their own honey reserves. At the beginning of the overwintering period, approximately 15 kg of honey was provided per colony. When the initial reserves were exhausted, additional honey was supplied in approximately 1 kg portions per colony as required. Total winter feed consumption was determined from the initial amount of honey provided and any additional honey supplied during the overwintering period.

Colony-level values were used in subsequent statistical analyses. For traits evaluated using the four-point scoring system, higher scores consistently represented more favorable colony-performance characteristics.

### 2.5. Morphometric Analysis

Ten worker bees were randomly collected from each experimental colony for wing morphometric analysis. The right forewing of each worker was carefully removed using fine forceps. The wings were mounted flat on transparent adhesive tape, arranged without overlap, and covered with a second transparent layer to maintain their position during image acquisition. The prepared wing samples were scanned to obtain digital images suitable for morphometric analysis.

The mounted wings were scanned using an Epson Perfection V600 Photo scanner (Seiko Epson Corp., Suwa, Nagano, Japan) at a resolution of 3200 dpi, producing images of 5782 × 3946 pixels. Identical scanning settings were used for all specimens to maintain a consistent image scale and resolution. No separate physical scale calibration was required for calculation of the cubital and Hantel indices because these parameters are based on ratios of wing-vein measurements rather than absolute linear dimensions. Wing images were digitized by identifying the required vein-junction landmarks, and the resulting landmark-coordinate files were processed using MorphoXL software (version 1.0). To minimize operator-related measurement error, landmark placement was performed by the same trained operator for all specimens using an identical predefined landmark configuration. Each image was visually inspected before analysis, and landmark positions were checked for correct placement at the corresponding vein junctions. Wings with folding, distortion, damaged venation, or insufficiently clear vein junctions were excluded from measurement. When landmark placement was uncertain, the image was re-examined and the landmark position was corrected before the morphometric indices were calculated. MorphoXL was used to calculate the cubital index, Hantel index, and discoidal shift for each wing. Colony-level values were calculated from the individual worker measurements. Discoidal shift was quantified as an angular measurement (discoidal shift angle, °) based on the wing-vein landmarks implemented in MorphoXL. Thus, the values reported in this study represent degrees and should not be interpreted as the dimensionless or normalized discoidal-shift values commonly reported on a scale close to zero.

### 2.6. Statistical Analysis

Statistical analyses were selected according to the measurement scale and specific objectives of the study. One-way analysis of variance (ANOVA) was used to test for differences among apiaries in continuous variables, including hygienic behavior, winter feed consumption, and wing morphometric traits. Ordinal colony-performance traits assessed on four-point scales, including calmness, gentleness, swarming tendency, overwintering performance, and productivity, were analyzed using the Kruskal–Wallis test. Principal component analysis (PCA) was used as a descriptive multivariate method to summarize covariance among the measured traits and to identify variables contributing to multivariate differentiation among colonies. Hierarchical cluster analysis (HCA) was used as a complementary exploratory method to visualize similarities among colonies based on their multivariate phenotypic profiles.

Statistical analyses were performed using JMP Pro 17 (SAS Institute Inc., Cary, NC, USA). The honey bee colony was considered the primary observational unit for analyses of hygienic behavior and colony performance (*n* = 50 colonies). For morphometric traits, measurements obtained from individual workers within each colony were averaged to obtain colony-level values before their inclusion in colony-level analyses. Thus, individual worker bees were not treated as independent replicates in analyses relating morphometric traits to hygienic behavior.

For continuous variables, assumptions of normality and homogeneity of variance were evaluated using the Shapiro–Wilk test applied to model residuals and Levene’s test, respectively. Differences among apiaries were evaluated using one-way ANOVA, followed by Tukey’s HSD test when the overall effect was significant. Ordinal colony-performance traits were analyzed using the Kruskal–Wallis test. When a significant overall difference was detected, appropriate non-parametric pairwise comparisons with adjustment for multiple testing were performed. Statistical significance was set at *p* < 0.05.

PCA was performed using standardized colony-level variables to account for differences in measurement scales among hygienic behavior, colony-performance traits, and morphometric characteristics. The first principal components were retained to summarize the major patterns of covariance among traits, and variable loadings were used to interpret their contributions to the principal-component axes. PCA was used for descriptive multivariate exploration and not for causal inference.

Hierarchical cluster analysis was performed using Ward’s agglomerative method with Euclidean distance on standardized colony-level variables. This analysis was used to visualize phenotypic similarities and grouping patterns among the studied colonies and was interpreted as an exploratory descriptive analysis rather than as evidence of independent ecological effects.

To determine whether the association between cubital index and hygienic behavior represented a consistent colony-level relationship within apiaries or primarily reflected differences among apiaries, the cubital index was decomposed into within-apiary and between-apiary components. The within-apiary component was obtained by centering each colony-level cubital-index value around the corresponding apiary mean. Hygienic behavior (%) was then modeled as the dependent variable, with the within-apiary cubital-index component included as a fixed effect and apiary included as a random intercept. This analysis specifically tested whether colonies with relatively higher cubital-index values than other colonies within the same apiary also exhibited higher hygienic behavior. Statistical significance was set at *p* < 0.05.

## 3. Results

### 3.1. Hygienic Behavior

The assumptions of one-way ANOVA were satisfied. The residuals did not significantly deviate from normality (Shapiro–Wilk W = 0.974, *p* = 0.330), and Levene’s test indicated homogeneity of variances among the five apiaries (F(4,45) = 0.832, *p* = 0.512).

The highest mean brood-removal efficiencies were recorded in the Nurik (96.62 ± 1.37%) and KazNARU (95.78 ± 1.96%) apiaries, both located in the foothill ecological category. Colonies from the Zhusip apiary, located in the steppe category, showed a similarly high mean brood-removal efficiency (94.85 ± 1.90%), although the apiary mean was slightly below the predefined ≥95% threshold for classification as highly hygienic. Lower mean brood-removal efficiency was observed in the Med Ainabekov apiary (90.11 ± 2.29%), while the lowest value was recorded in the Baizhigitov apiary (79.90 ± 1.88%).

Tukey’s HSD post hoc test identified significant pairwise differences among the studied apiaries (*p* < 0.05; Table 2). Overall, the results demonstrate substantial variation in hygienic behavior among the five apiaries. Although the observed differences showed some correspondence with the ecological conditions of the study sites, they should not be interpreted as independent effects of ecological zone because apiary identity, management practices, and honey bee population background were not fully separated in the present study design.

### 3.2. Colony-Performance Traits

Colony-performance traits varied among the five studied apiaries (Table 3).

Kruskal–Wallis tests revealed significant differences among apiaries for calmness (H = 31.17, *p* < 0.001), gentleness (H = 35.45, *p* < 0.001), swarming tendency (H = 24.89, *p* < 0.001), overwintering performance (H = 33.52, *p* < 0.001), and productivity (H = 26.97, *p* < 0.001). These results demonstrate substantial apiary-level variation in all ordinal colony-performance traits. Winter feed consumption, which was analyzed as a continuous variable, also differed among apiaries (Table 3).

### 3.3. Morphometric Characteristics

Morphometric characteristics differed significantly among the five studied apiaries (Table 4). The cubital index showed significant variation among apiaries [F(4,45) = 328.17, *p* < 0.001], with the highest mean value recorded in the Nurik apiary (2.84 ± 0.04) and the lowest in the Baizhigitov apiary (2.13 ± 0.05). Intermediate values were observed in the KazNARU (2.54 ± 0.04), Zhusip (2.46 ± 0.06), and Med Ainabekov (2.33 ± 0.04) apiaries (Table 4).

The Hantel index also differed significantly among apiaries [F(4,45) = 37.64, *p* < 0.001], ranging from 0.97 ± 0.02 in Baizhigitov to 1.07 ± 0.03 in KazNARU. Significant differences were also observed for the discoidal shift angle [F(4,45) = 366.31, *p* < 0.001]. The highest mean discoidal shift angle was recorded in KazNARU (4.02 ± 0.17), followed by Nurik (3.46 ± 0.15), Med Ainabekov (2.86 ± 0.23), Zhusip (2.07 ± 0.13), and Baizhigitov (1.08 ± 0.29).

Overall, these results demonstrate substantial morphometric variation among colonies from the five studied apiaries. Although some of this variation corresponded with the ecological categories of the study sites, the observed differences should not be interpreted as independent effects of ecological conditions because apiary identity, honey bee population background, and other apiary-level factors were not fully separated in the present study design.

### 3.4. Multivariate Analysis

PCA summarized the relationships among hygienic behavior, colony-performance traits, and morphometric characteristics (Figure 2). The first two principal components explained 73.8% of the total variance, with PC1 accounting for 58.9% and PC2 for 14.9%.

The PCA biplot revealed differentiation among colonies from the five studied apiaries, with colonies from some apiaries occupying similar regions of the multivariate space. Colonies from the Nurik and KazNARU apiaries (Foothill) and the Zhusip apiary (Steppe) were generally positioned on the positive side of PC1 and were associated with higher hygienic behavior, greater gentleness, greater calmness, higher cubital index values, and increased winter feed consumption. In contrast, colonies from the Baizhigitov apiary (Mountain) were positioned predominantly on the negative side of PC1 and were characterized by lower hygienic behavior and lower values of several morphometric and colony-performance traits. Colonies from the Med Ainabekov apiary (Mountain) occupied a comparatively intermediate position in the multivariate space.

Among the measured variables, hygienic behavior, gentleness, calmness, cubital index, and productivity contributed most strongly to differentiation along PC1, whereas winter hardiness and winter feed consumption primarily contributed to variation along PC2. Overall, the PCA indicates multivariate differentiation among colonies associated with their apiary of origin; however, this descriptive pattern should not be interpreted as evidence of an independent causal effect of ecological zone.

### 3.5. Hierarchical Cluster Analysis

Hierarchical cluster analysis (Ward’s method) was performed using hygienic behavior, colony-performance traits, and morphometric characteristics to evaluate phenotypic similarity among the investigated honey bee colonies (Figure 3). The resulting dendrogram revealed distinct grouping patterns among colonies from the five studied apiaries. Colonies from the Nurik and KazNARU apiaries (Foothill) and the Zhusip apiary (Steppe) showed relatively similar multivariate profiles and tended to group more closely. In contrast, colonies from the Baizhigitov apiary (Mountain) formed a more distinct group, whereas colonies from the Med Ainabekov apiary (Mountain) occupied a comparatively intermediate position. This overall grouping pattern was broadly consistent with the differentiation observed in the PCA biplot.

The accompanying heatmap further illustrated differences in standardized trait values among individual colonies. Colonies from the Nurik, KazNARU, and Zhusip apiaries were generally characterized by higher hygienic behavior, greater gentleness, higher calmness scores, and higher cubital index values. In contrast, colonies from the Baizhigitov apiary generally exhibited lower values for several of these traits, whereas colonies from the Med Ainabekov apiary showed comparatively intermediate phenotypic characteristics. Overall, the hierarchical clustering analysis demonstrated phenotypic grouping among the studied colonies associated with apiary of origin. As a descriptive multivariate analysis, however, this clustering should not be interpreted as evidence of an independent causal effect of ecological conditions.

### 3.6. Apiary-Level Effects on the Relationship Between Cubital Index and Hygienic Behavior

The pooled analysis showed a strong positive association between cubital index and hygienic behavior. However, after separating within-apiary variation from differences among apiaries, the within-apiary association was not statistically significant (β = 9.42, 95% CI: −2.82 to 21.65, *p* = 0.128). This indicates that colonies with relatively higher cubital-index values within a given apiary did not consistently show greater hygienic behavior. Therefore, the strong overall association was primarily attributable to differences among apiaries rather than to a consistent colony-level biological relationship (Table 5).

## 4. Discussion

Hygienic behavior differed substantially among the five studied apiaries, with the highest brood-removal efficiencies observed in Nurik and KazNARU and the lowest in Baizhigitov. These differences are consistent with previous studies demonstrating substantial environmental and population-related variation in hygienic behavior [1]. Against a backdrop of elevated colony losses driven by a complex of parasites, pathogens, pesticides and environmental stressors [45], the parasitic mite *Varroa destructor*, together with its vectored viruses, remains the single most damaging biotic threat to *A. mellifera* [46,47]. Because the sustainable, treatment-free management of this parasite ultimately depends on the bees’ own defences, our study set out to characterise hygienic behaviour—a heritable form of social immunity first described by Rothenbuhler [6]—and its associations with colony-performance and morphometric traits in honey bee populations from Kazakhstan, where previous studies have documented substantial morphometric and population diversity [44,48,49].

The landmark pan-European COLOSS genotype–environment interaction experiment, which monitored 621 colonies of 16 genotypes from five subspecies across 21 test apiaries in 2010–2011, concluded that “genotype and location have a significant effect on the analysed traits,” including hygienic, swarming and defensive behaviour [29]. The companion survival analysis (2009–2011) found that “the survival of bee colonies with queens of local origin was statistically significantly higher than colonies with non-local queens” [41,50], demonstrating strong genotype-by-environment interactions [19]. A strong positive association between cubital index and hygienic behavior was observed when all colonies were analyzed together. However, this relationship was not statistically significant in the within-apiary analysis (β = 9.42, 95% CI: −2.82 to 21.65, *p* = 0.128). This finding suggests that the pooled relationship largely reflected differences among apiaries or honey bee population backgrounds rather than a consistent colony-level biological association. Because cubital index is widely used as an indicator of honey bee population and subspecies structure, variation among apiaries in population composition may have contributed to the apparent overall relationship with hygienic behavior. Accordingly, cubital index should not be interpreted as a direct predictor of hygienic behavior based on the present dataset.

The variation observed among the studied apiaries showed some correspondence with previously reported altitudinal patterns of hygienic behavior. The highest mean brood-removal efficiencies were recorded in the Nurik and KazNARU apiaries (Foothill; 96.62% and 95.78%, respectively) and in the Zhusip apiary (Steppe; 94.85%), whereas the two Mountain apiaries differed substantially, with mean removal efficiencies of 90.11% in Med Ainabekov and 79.90% in Baizhigitov. These observations are broadly consistent with studies reporting variation in hygienic behavior across altitude. In the central highlands of Ecuador, hygienic behavior measured using the pin-killed brood assay averaged 80% ± 9.7% across 75 colonies sampled at three elevation ranges, with higher hygienic performance reported at lower altitudes [51]. That our mountain apiary fell below, and the foothill and steppe apiaries met or approached, the widely used “highly hygienic” classification threshold is significant for practical selection. The foothill and steppe apiaries met or approached the commonly used threshold of approximately 95% brood removal within 24 h for identifying highly hygienic colonies. Spivak and Danka [7] reported that colonies removing 95% of freeze-killed brood within 24 h were considered highly hygienic. These comparisons indicate that colonies from several of the studied apiaries exhibited high levels of general hygienic brood-removal behavior, although confirmation across additional apiaries, seasons, and genetic backgrounds is required before their suitability for breeding can be established.

Methodologically, our use of the pin-killed brood assay follows a well-established approach for evaluating general hygienic brood-removal behavior. The pin-kill bioassay was introduced as a simplified screening method for behavioral resistance to American foulbrood [18], and killed-brood assays have subsequently been widely used to assess hygienic behavior in honey bee colonies [7,20]. The pin-killed brood assay provides a standardized measure of general hygienic brood-removal behavior; however, it does not directly quantify VSH, mite reproductive success, pathogen burden, or colony-level resistance to Varroa [7,52]. Previous studies have reported low-to-moderate, and in some populations higher, heritability estimates for hygienic behavior, indicating that this trait can respond to selection, although the between-apiary differences observed in the present study cannot be attributed solely to genetic effects: estimates range from h^2^ = 0.65 (Harbo & Harris, USDA-ARS Baton Rouge) [24] and 0.18–0.36 in earlier work [53] to lower values in free-mated populations, while genetic-parameter studies in Canadian and Swiss stocks place hygienic behaviour at low-to-moderate heritability with positive or null genetic correlations to other economically important traits [54,55]. This latter point is reassuring for breeders and central to the Varroa-resistance breeding programmes established in Europe [22] and North America [28] and to the catalogue of naturally mite-surviving and selected resistant populations reviewed recently [52,56].

A particularly novel element of our results is the positive, significant association between the cubital index—a wing-venation morphometric trait—and hygienic behaviour, complemented by measurement of the Hantel index and discoidal shift. Wing-venation indices and geometric morphometrics are primarily established for discriminating *A. mellifera* subspecies and evolutionary lineages and for detecting hybridisation, performing comparably to microsatellite and mtDNA markers [33,34,35]. Their direct correlation with a behavioural trait is rarely reported, but not without precedent: significant genetic correlations exist between honey bee behavioural characteristics and forewing length [57], and numerous morphometric characters correlate—albeit inconsistently across populations—with colony defensive behaviour. Our finding therefore adds to the limited evidence concerning associations between wing morphometry and hygienic behavior. Because the cubital index differs among honey bee subspecies and populations, and because Kazakh colonies comprise *A. m. carnica* alongside local and admixed stock, the association observed in the present study may partly reflect underlying population/subspecies structure covarying with hygienic performance rather than a direct functional relationship between wing morphology and brood-removal behavior. Differences in hygienic behavior among honey bee races have previously been reported; for example, Italian colonies showed greater brood-removal activity than Carniolan colonies in pin-killed brood and artificial-infestation assays [58]. Genetic and morphometric studies of honey bees in Kazakhstan have also documented substantial population diversity and variation among sampled populations [44,49]. Therefore, the association between cubital index and hygienic behavior observed here should be considered preliminary and requires further validation while controlling for genetic background and apiary-level environmental and management factors.

Winter feed consumption is a heritable trait incorporated into northern breeding indices [54], and colonies that overwinter economically while remaining strong are generally those best adapted to local conditions [37,41]. Winter feed consumption differed substantially among the studied apiaries; however, it was not independently associated with hygienic behavior in the mixed-effects model (β = −0.13, *p* = 0.682). Therefore, the observed between-apiary differences in winter feed consumption are more appropriately interpreted in relation to apiary-specific overwintering conditions and management rather than hygienic behavior [50,54]. Because published genetic-parameter work indicates hygienic behaviour is not antagonistically correlated with honey yield or gentleness, simultaneous selection for hygiene, gentleness, productivity and overwintering economy is feasible [54,55]. The grouping patterns observed in the principal component analysis and Ward’s hierarchical cluster analysis further demonstrated multivariate phenotypic differentiation among colonies from the studied apiaries based on behavioral, performance, and morphometric traits [44,48].

Several limitations should be considered when interpreting these findings. Only five apiaries were sampled, and ecological conditions were therefore partially confounded with apiary identity, management practices, and the honey bee population background represented at each site. Detailed information on queen pedigree and genetic origin was not available for all experimental colonies, and environmental factors such as temperature and forage availability were not systematically quantified. Although the revised mixed-effects analysis accounts for the clustering of colonies within apiaries, it cannot fully disentangle these apiary-level factors or establish independent effects of ecological conditions. Hygienic behavior was assessed using a single-time-point pin-killed brood assay, which, although widely used for evaluating general brood-removal behavior, does not directly quantify VSH, *Varroa destructor* infestation, pathogen load, or colony-level disease resistance. Furthermore, the observed association between cubital index and hygienic behavior is correlational and may partly reflect underlying population structure rather than a direct functional relationship between wing morphology and brood-removal behavior.

Future studies should therefore (i) include multiple independent apiaries within each ecological category and repeated sampling across seasons and years; (ii) incorporate standardized environmental and management variables, including altitude, temperature, forage availability, supplementary feeding, and *Varroa*-control practices; (iii) complement the pin-killed brood assay with direct measurements of VSH, mite infestation, pathogen load, and colony survival; (iv) integrate molecular genetic markers with morphometric analyses to investigate the population/subspecies basis of the cubital-index association; and (v) evaluate promising colonies through replicated breeding trials across contrasting environments to better partition genetic and environmental contributions. Such studies will be necessary to validate the present associations before hygienic behavior and associated phenotypic traits can be reliably incorporated into future honey bee breeding programs in Kazakhstan.

## 5. Conclusions

This study demonstrated substantial variation in hygienic behavior among honey bee colonies from five apiaries in Kazakhstan, with the highest brood-removal efficiencies observed in the Nurik, KazNARU, and Zhusip apiaries and the lowest in the Baizhigitov apiary. Hygienic behavior was associated with variation in colony-performance and wing-morphometric characteristics, and the linear mixed-effects analysis identified a significant positive association between cubital index and hygienic behavior. However, this relationship should be interpreted as an association rather than evidence of a direct functional effect, because morphometric variation may reflect underlying population structure.

These findings provide baseline information for the characterization of hygienic behavior in honey bee populations of Kazakhstan. Future studies should include multiple independent apiaries within each ecological category, repeated evaluations across seasons and years, standardized environmental and management variables, and molecular characterization of honey bee populations. Combining the pin-killed brood assay with direct measurements of VSH, *Varroa destructor* infestation, pathogen load, and colony survival will be important for determining whether the observed phenotypic associations can be reliably incorporated into future honey bee breeding programs in Kazakhstan.

## Figures and Tables

**Figure 1:**
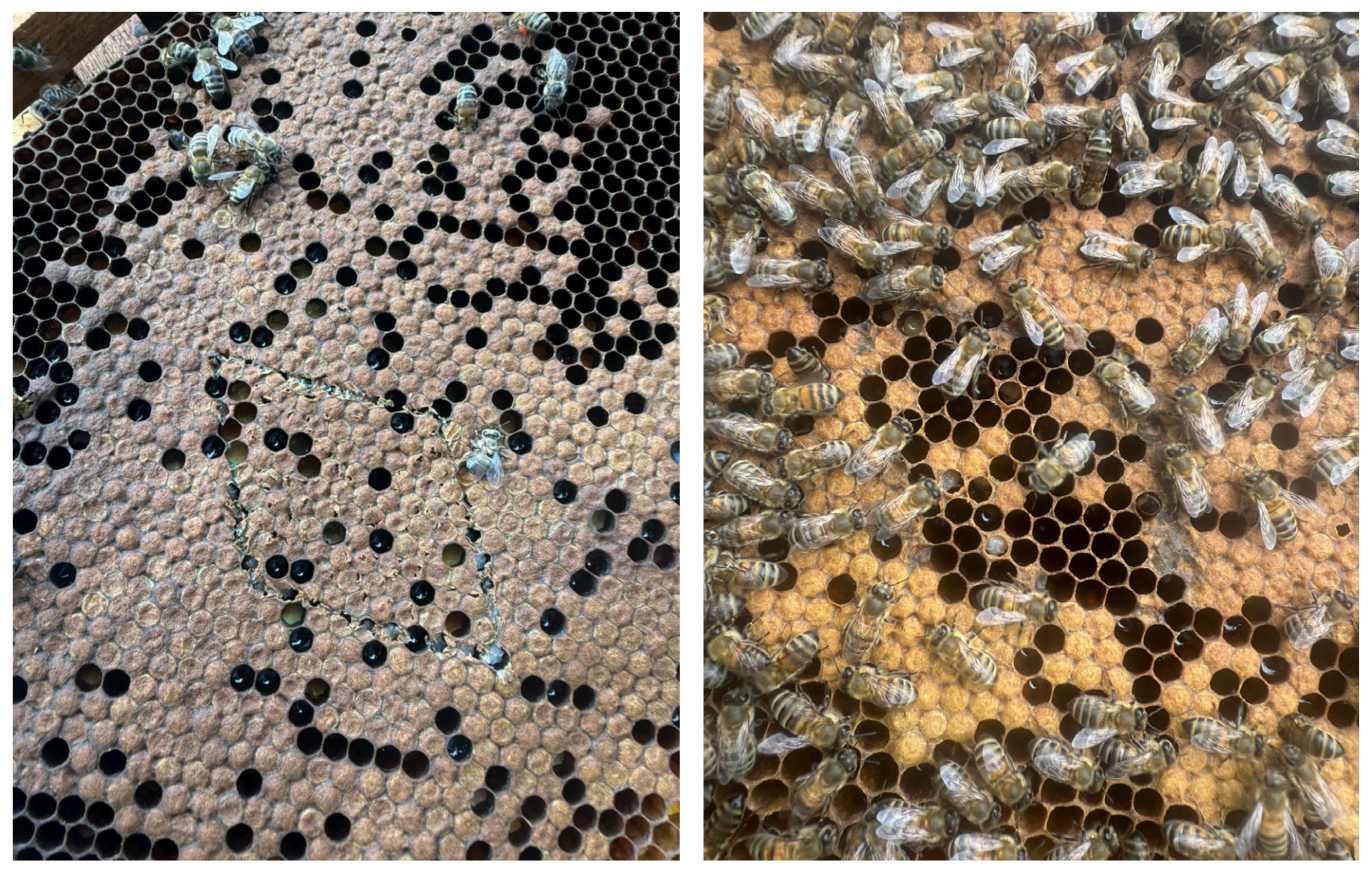
Assessment of Hygienic Behavior Using the Pin-Killed Brood Assay.

**Figure 2:**
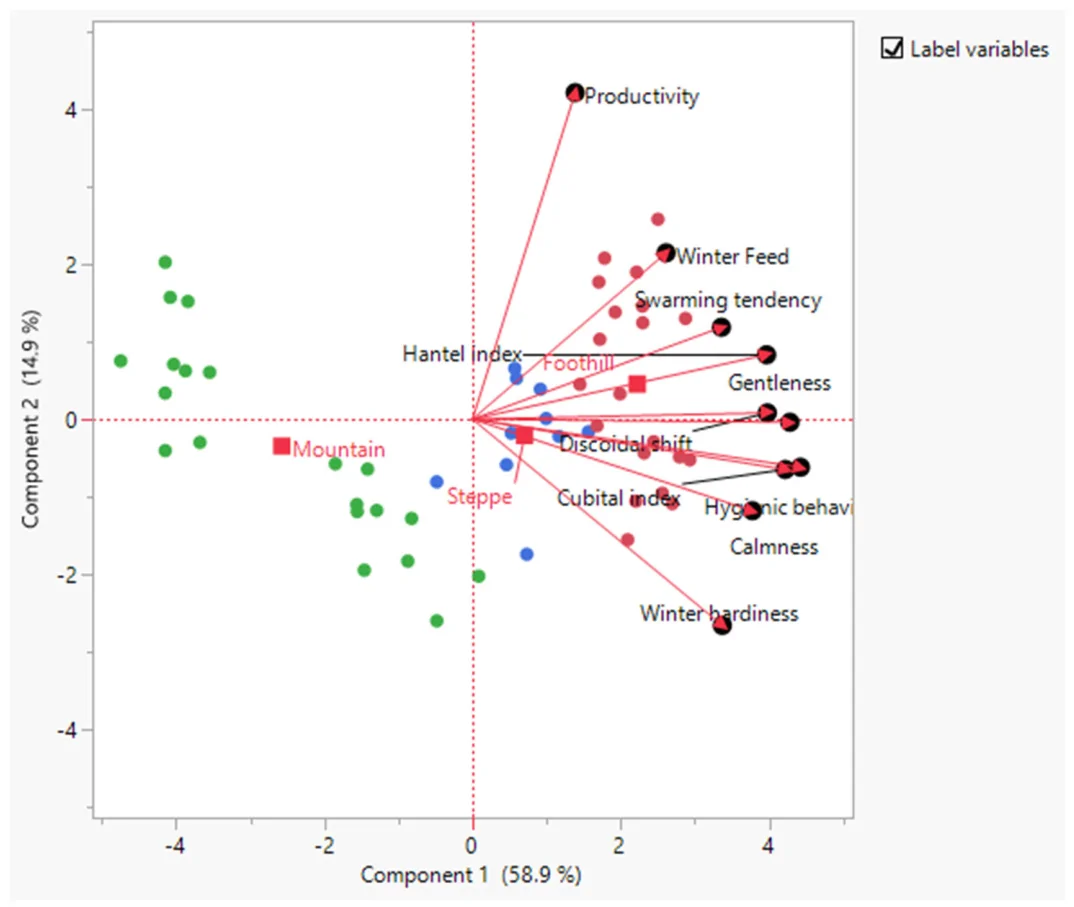
PCA biplot based on standardized colony-level hygienic behavior, colony-performance traits (Calmness, gentleness, swarming tendency, overwintering performance, productivity, and winter feed consumption), and wing morphometric traits (cubital index, Hantel index, and discoidal shift). Variables were standardized before PCA to account for differences in measurement scales. Arrows represent variable loadings, with their direction and length indicating the direction and relative contribution of each variable to the principal components. Individual points represent honey bee colonies and are identified according to the apiary of origin. PC1 and PC2 explain 58.9% and 14.9% of the total variance, respectively.

**Figure 3:**
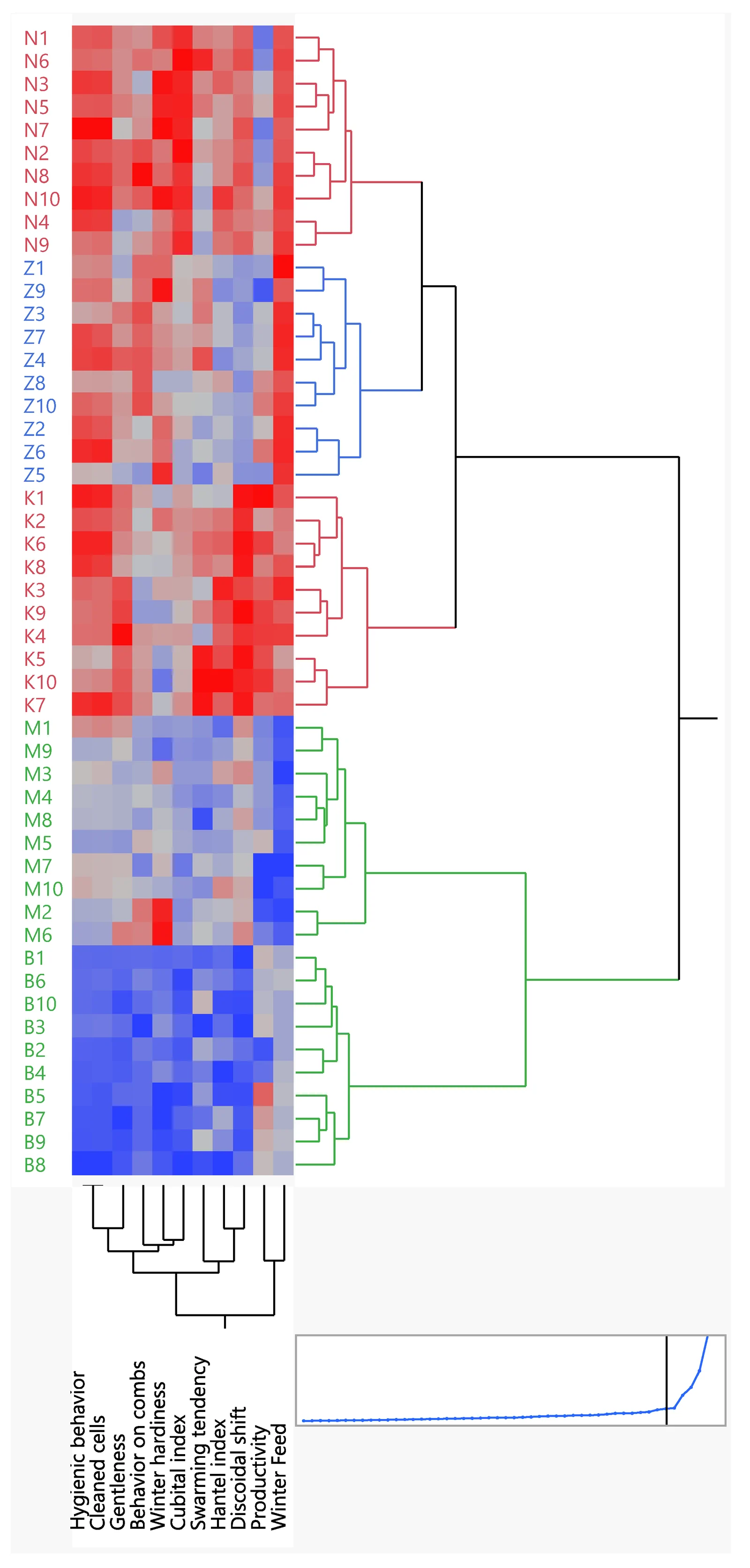
Hierarchical Cluster Analysis (Ward’s Method) of Honey Bee Colonies Based on Hygienic Behavior, Colony-Performance Traits, and Morphometric Characteristics.

**Table 1:** Characteristics of the Experimental Apiaries.

No.	Apiary	Total Colonies	Production Direction	Latitude	Longitude	Sea Level (m a.s.l.)
1	Nurik	245	Honey and breeding	43.1338° N	75.8420° E	1071
2	KazNARU	48	Honey and breeding	43.4417° N	77.3359° E	740
3	Med Ainabekov”	1510	Honey, breeding, bee packages	41.7601° N	69.3872° E	1375
4	Baizhigitov	223	Honey, breeding, bee packages	42.9749° N	75.5645° E	1530
5	Zhusip”	545	Honey and breeding	41.7545° N	69.3848° E	250

“Total colonies” refers to the total number of honey bee colonies maintained at each respective apiary, not the number of colonies included in the study. Ten colonies were sampled from each apiary (*n* = 50 colonies in total).

**Table 2:** Hygienic Behavior of Honey Bee Colonies Assessed by the Pin-Killed Brood Assay.

Apiary	Ecological Zone	Hygienic Behavior (%)	Range, %
Nurik	Foothill	96.62 ± 1.37 ^a^	94.6–98.9
KazNARU	Foothill	95.78 ± 1.96 ^a^	92.5–98.2
Med Ainabekov	Mountain	90.11 ± 2.29 ^b^	86.7–93.5
Baizhigitov	Mountain	79.90 ± 1.88 ^c^	75.9–82.6
Zhusip	Steppe	94.85 ± 1.90 ^a^	92.1–97.6
ANOVA F(4,45)		145.7	
*p*-value		<0.001	

Values are presented as mean ± SD. Range indicates the minimum and maximum colony-level hygienic behavior values. Different superscript letters indicate significant differences among apiaries according to Tukey’s HSD test (*p* < 0.05).

**Table 3:** Colony-Performance Traits of Honey Bee Colonies.

Apiary	Calmness	Gentleness	Swarming Tendency	Winter Hardiness	Productivity	Winter Feed (kg)
Nurik	3.78 [3.77–3.85] ^a^	3.70 [3.62–3.73] ^ab^	3.73 [3.69–3.80] ^ab^	3.87 [3.83–3.96] ^a^	3.58 [3.52–3.67] ^a^	19.62 ± 0.32 ^ab^
KazNARU	3.75 [3.70–3.77] ^a^	3.78 [3.74–3.81] ^a^	3.80 [3.72–3.92] ^a^	3.72 [3.66–3.76] ^bc^	3.84 [3.78–3.86] ^b^	18.95 ± 0.91 ^a^
Med Ainabekov	3.67 [3.63–3.72] ^ab^	3.58 [3.54–3.61] ^bc^	3.63 [3.61–3.67] ^bc^	3.73 [3.68–3.78] ^ab^	3.51 [3.43–3.55] ^a^	8.41 ± 0.91 ^d^
Baizhigitov	3.52 [3.51–3.54] ^b^	3.29 [3.26–3.33] ^c^	3.60 [3.54–3.65] ^c^	3.51 [3.45–3.56] ^c^	3.64 [3.60–3.66] ^a^	15.16 ± 0.66 ^c^
Zhusip	3.85 [3.76–3.87] ^a^	3.65 [3.61–3.68] ^ab^	3.72 [3.70–3.79] ^ab^	3.83 [3.78–3.85] ^ab^	3.62 [3.57–3.70] ^a^	20.23 ± 0.59 ^b^
Test statistic	H = 31.17	H = 35.45	H = 24.89	H = 33.52	H = 26.97	F(4,45) = 476.09
*p*-value	<0.001	<0.001	<0.001	<0.001	<0.001	<0.001

Ordinal colony-performance traits are presented as median [interquartile range, IQR] (*n* = 10 colonies per apiary) and were compared among apiaries using the Kruskal–Wallis test. Following a significant overall test, pairwise comparisons were performed using Dunn’s test with Holm adjustment for multiple comparisons. Different superscript letters within the same column indicate significant pairwise differences (adjusted *p* < 0.05); values sharing at least one superscript letter do not differ significantly. Winter feed consumption is presented as mean ± SD and was analyzed using one-way ANOVA followed by Tukey’s HSD test.

**Table 4:** Morphometric Characteristics of Worker Honey Bees.

Apiary	Cubital Index	Hantel Index	Discoidal Shift (°)
Nurik	2.84 ± 0.04 ^a^	1.06 ± 0.02 ^a^	3.46 ± 0.15 ^a^
KazNARU	2.54 ± 0.04 ^b^	1.07 ± 0.03 ^a^	4.02 ± 0.17 ^b^
Med Ainabekov	2.33 ± 0.04 ^c^	1.01 ± 0.02 ^b^	2.86 ± 0.23 ^c^
Baizhigitov	2.13 ± 0.05 ^d^	0.97 ± 0.02 ^c^	1.08 ± 0.29 ^d^
Zhusip	2.46 ± 0.05 ^b^	1.01 ± 0.02 ^b^	2.07 ± 0.13 ^e^
ANOVA, F(4,45)	328.17	37.64	366.31
*p*-value	<0.001	<0.001	<0.001

Values are presented as mean ± SD (*n* = 10 colonies per apiary). Differences among apiaries were evaluated using one-way ANOVA. When the overall ANOVA was significant, pairwise comparisons were performed using Tukey’s HSD test. Different superscript letters within each column indicate significant differences among apiaries according to Tukey’s HSD test (*p* < 0.05).

**Table 5:** Within-apiary association between cubital index and hygienic behavior.

Effect	Estimate (β)	95% CI	*p*
Within-apiary cubital index	9.42	−2.82 to 21.65	0.128

Hygienic behavior (%) was the dependent variable. Cubital index was centered within apiary to separate within-apiary variation from between-apiary differences. Apiary was included as a random intercept. Statistical significance was set at *p* < 0.05.

## Data Availability

The data presented in this study are available from the corresponding author upon reasonable request.

## Abbreviations

The following abbreviations are used in this manuscript:

ANOVA	Analysis of Variance
GLM	General Linear Model
HCA	Hierarchical Cluster Analysis
HSD	Honestly Significant Difference
PCA	Principal Component Analysis
VSH	Varroa-Sensitive Hygiene
